# Meiotic Studies on Combinations of Chromosomes With Different Sized Centromeres in Maize

**DOI:** 10.3389/fpls.2018.00785

**Published:** 2018-06-13

**Authors:** Fangpu Han, Jonathan C. Lamb, Morgan E. McCaw, Zhi Gao, Bing Zhang, Nathan C. Swyers, James A. Birchler

**Affiliations:** ^1^Division of Biological Sciences, University of Missouri, Columbia, MO, United States; ^2^State Key Laboratory of Plant Cell and Chromosome Engineering, Institute of Genetics and Developmental Biology, Chinese Academy of Sciences, Beijing, China

**Keywords:** centromere misdivision, centromere competition, sister chromatids, recombination, B chromosome, meiotic drive

## Abstract

Multiple centromere misdivision derivatives of a translocation between the supernumerary B chromosome and the short arm of chromosome 9 (TB-9Sb) permit investigation of how centromeres of different sizes behave in meiosis in opposition or in competition with each other. In the first analysis, heterozygotes were produced between the normal TB-9Sb and derivatives of it that resulted from centromere misdivision that reduced the amounts of centromeric DNA. These heterozygotes could test whether these drastic differences would result in meiotic drive of the larger chromosome in female meiosis. Cytological determinations of the segregation of large and small centromeres among thousands of progeny of four combinations were made. The recovery of the larger centromere was at a few percent higher frequency in two of four combinations. However, examination of phosphorylated histone H2A-Thr133, a characteristic of active centromeres, showed a lack of correlation with the size of the centromeric DNA, suggesting an expansion of the basal protein features of the kinetochore in two of the three cases despite the reduction in the size of the underlying DNA. In the second analysis, plants containing different sizes of the B chromosome centromere were crossed to plants with TB-9Sb with a foldback duplication of 9S (TB-9Sb-Dp9). In the progeny, plants containing large and small versions of the B chromosome centromere were selected by FISH. A meiotic “tug of war” occurred in hybrid combinations by recombination between the normal 9S and the foldback duplication in those cases in which pairing occurred. Such pairing and recombination produce anaphase I bridges but in some cases the large and small centromeres progressed to the same pole. In one combination, new dicentric chromosomes were found in the progeny. Collectively, the results indicate that the size of the underlying DNA of a centromere does not dramatically affect its segregation properties or its ability to progress to the poles in meiosis potentially because the biochemical features of centromeres adjust to the cellular conditions.

## Introduction

The centromere is the part of the chromosome that organizes the kinetochore for movement functions. In any one species, there is typically characteristic DNA repeats at the primary constriction but it is not clear to what extent this DNA functions in determining the position of the kinetochore. For example, in maize, there are two typical repetitive DNA elements at centromeres, namely the 156 base pair unit satellite, CentC, and an active retrotransposon, Centromere Retrotransposon Maize, CRM (Ananiev et al., 1998; Zhong et al., 2002; Nagaki et al., 2003; Schneider et al., 2016). Nevertheless, inactive centromeres have been described in maize that still contain these sequences (Han et al., 2006; Gao et al., 2011) and in addition the occurrence of *de novo* centromere formation over unique sequences on maize chromosomal fragments has been shown to occur in several examples (Topp et al., 2009; Fu et al., 2013; Zhang et al., 2013; Liu et al., 2015). Thus, the DNA does not appear to be necessary or sufficient to establish the site of the kinetochore. The consistent biochemical feature of active centromeres in maize is the presence of a Histone 3 variant called CENH3 (Zhong et al., 2002) and other associated proteins. They are missing in the inactive centromeres and present at the *de novo* sites (Han et al., 2006; Gao et al., 2011; Fu et al., 2013; Liu et al., 2015).

The centromere sequences evolve quickly and are exchanged across the genome rapidly in evolutionary time. In contrast, the biochemical machinery of the kinetochore is highly conserved. This difference has been referred to as the centromere paradox (Henikoff et al., 2001). One possible scenario to explain this paradox is that the histone associated with the centromeric DNA, CENH3, is in an evolutionary conflict with the associated DNA. Expansion or contraction of the DNA cluster might cause a greater recovery of the larger DNA array through female meiosis via its progression to the basal megaspore, which is the only product of meiosis in the female gametophyte that is passed to the next generation. Since this hypothesis was formulated, there has been accumulating evidence for an epigenetic aspect to centromere specification as evidenced by numerous cases of inactive centromeres as well as *de novo* centromere formation over unique DNA, as noted above. Such events have been documented in various inbred lines of maize with the homogenization of centromeres being accomplished by preferential insertion of the common elements into centromeric chromatin (Schneider et al., 2016). Nevertheless, the availability of a collection of chromosomes that are all derived from a common progenitor but having drastically reduced amounts of centromeric DNA would allow a test of whether centromere size affects the frequency of segregation between the two sizes of centromeres.

In previous studies in our laboratory, a collection of reduced sized centromeres was produced via misdivision of a particular centromere (Kaszas and Birchler, 1996, 1998). Centromere misdivision results when there is attachment of a single kinetochore to both poles that sever the chromosome at the primary constriction but for which both products are capable of function (Carlson, 1970; Carlson and Chou, 1981). The particular centromere involved is that of the supernumerary B chromosome that is present in a translocation between a B chromosome and the short arm of chromosome 9 (TB-9Sb). This chromosome arm has been used extensively in maize genetics because it contains several useful phenotypic markers. The B chromosome is a non-vital one that survives in maize lines by an accumulation mechanism consisting of non-disjunction at the second pollen mitosis (Roman, 1947) with the sperm containing the two B chromosomes preferentially fertilizing the egg in the process of double fertilization (Roman, 1948). The centromere of TB-9Sb was shown to undergo misdivision by Carlson (1970). Subsequently a large collection of misdivision derivatives was recovered and studies of their molecular features demonstrated a progressive reduction in underlying DNA sequences (Kaszas and Birchler, 1996, 1998) that were fractured at the centromeric core (Jin et al., 2005). The B chromosome centromere has an advantage for centromere studies because it contains a specific repeat sequence in and around its centromere (Alfenito and Birchler, 1993; Jin et al., 2005). This collection of different sized centromeres on the same chromosome placed in heterozygotes with the progenitor chromosome with a normal sized centromere was examined in the present study for any evidence of differential segregation or meiotic drive of the divergently sized centromeres.

In addition to segregation properties of large and small centromeres, the collection allowed a determination of segregation strength against the progenitor centromere by using a modified form of TB-9Sb that contains a reverse duplication. Barbara McClintock generated a chromosome that has a duplication of most of 9S but in reverse order (McClintock, 1939, 1941). She used this chromosome to study the breakage-fusion-bridge (B-F-B) cycle because recombination within its limits would generate a dicentric chromosome that would break and initiate the cycle. This duplication was recombined onto TB-9Sb by Zheng and colleagues (Zheng et al., 1999) to produce TB-9Sb-Dp9 to study the chromosome type of B-F-B cycle. By producing heterozygotes of this chromosome with selected misdivision derivatives with reduced sized centromeres, recombination can occur between the derivative and the reversely oriented portion of TB-9Sb-Dp9 to form a dicentric with large and small centromeres. A previous study with one such derivative found dicentric chromosomes in the progeny in which the large centromere was active but the small centromere was inactive (Han et al., 2009). Here we report the results with other misdivision derivatives with regard to the ability to recombine and to the strength of the segregation of the opposed large and small centromeres.

The collection of centromere misdivision derivatives of the same chromosome together with its normal progenitor provide a unique opportunity to examine whether changing the amount of centromeric DNA has an impact on the segregation property or on the strength of its segregation. Interestingly, an examination of a biochemical feature of active centromeres, phosphorylation of histone H2A, revealed that the size of its signal was not always correlated with the relative amount of underlying DNA suggesting an adjustment of the functional size of the centromere similarly to what occurs when maize chromosomes are placed into oat (Wang et al., 2014). The results reveal that the amount of underlying DNA is not a reflection of the size of the biochemical foundation of kinetochores and there is little discernible effect of the size of the DNA array on segregation fidelity or strength.

## Materials and Methods

### Plant Materials

Homozygous stocks were produced of TB-9Sb and its misdivision derivatives Telocentric 3-5(+), Telo 2-2, Telo 4-5 and Telo 4-11 (**Table 1**) and confirmed cytologically. Crosses were performed to produce heterozygous combinations of the normal TB-9Sb and each of the misdivision chromosomes. Heterozygotes containing two copies of the 9-B chromosome and the normal and derivative B-9 chromosomes were grown to maturity for crosses made with pollen from a *c1 sh1 wx1* tester, which possesses three recessive mutations in 9S. In the progeny of such crosses, classifications of the segregation frequency were determined by FISH with the B chromosome specific sequence to identify the presence of the large or small centromere chromosome.

**Table 1:** Comparative size estimates of misdivision derivatives and the progenitor, TB-9Sb.

Chromosome	Estimated size of the B specific array (kb)
TB-9Sb	9000
Telo 4-11	2360
Telo 4-5	2180
Telo 2-2	2150
Telo 3-5(+)	1665
Telo 6-9	1160
Telo 4-4	490


*Sizes are based on restriction digests separated on CHEF gels and probed with the B chromosome specific centromeric repeat, which is present in and around the B chromosome centromere (Jin et al., 2005). Size estimates are derived from Kaszas and Birchler (1996, 1998).*

The chromosome, TB-9Sb-Dp9, was produced by Zheng et al. (1999) by recombining onto TB-9Sb a reverse duplication involving the short arm of chromosome 9 (McClintock, 1939, 1941). Telocentrics 2-2, 3-5(+), 4-4, 4-5, 4-11, and 6-9 (**Table 1**) have been described (Kaszas and Birchler, 1998). Seedlings of TB-9Sb-Dp9 heterozygous with telocentric chromosomes were identified by FISH of root tip cells. Male inflorescences in meiosis were collected from the heterozygotes and were fixed in ethanol: acetic-acid (3:1, v/v) on ice for 2 h, and transferred to 70% ethanol and stored at -20°C.

### DNA Probe Preparation

For classification of chromosomes in meiosis, the B-specific sequence (Alfenito and Birchler, 1993) was labeled with Texas-red-5-dUTP and knob heterochromatin sequence (Peacock et al., 1981) with fluorescein-12-dUTP, as described (Kato et al., 2004). In some cases, a labeled oligonucleotide probe of the telomere sequence was used to detect the B centromere due to cross hybridization with the B specific sequence (Alfenito and Birchler, 1993).

### Immunolocalization of H2AphThr133

Antibodies to H2AphThr133 were produced against peptides with a single phosphorylated Thr at position 133 (LPKK(pT)AEKA) of H2A as described (Su et al., 2017). Immunolocalization was performed as described (Han et al., 2009). Briefly, the samples were fixed in 4% paraformaldehyde for 2 h on ice. Then, the samples were treated with 1% Triton X-100 (1X PBS and 1 mM EDTA). The primary antibody was incubated overnight at 4 degrees followed by secondary antibody incubation at 37 degrees and DAPI staining. The primary and secondary antibodies were diluted in 3% BSA. For the immuno-FISH procedure, FISH was performed after immunolabeling. The concentrations of anti-H2Aph, anti-rabbit IgG and DAPI were 0.93 mg/ml, 0.015 mg/ml and 0.5 μg/ml, respectively.

### Meiotic Analysis

Meiotic images at various stages were collected from heterozygotes as described (Gao et al., 1999; Han et al., 2009).

## Results

### A Test of Meiotic Drive Between Large and Small Centromeres

The rationale to examine the issue of meiotic drive of different sized centromeres was to produce heterozygotes of TB-9Sb for which one copy is a misdivision derivative with a reduced size centromere and the other is the normal B chromosome centromere. The heterozygotes were then crossed as a female by a tester and the progeny were screened for the presence of the large and small centromeres, which are readily cytologically distinguishable. A skewed segregation ratio in the progeny would be indicative of meiotic drive.

**FIGURE 1:**
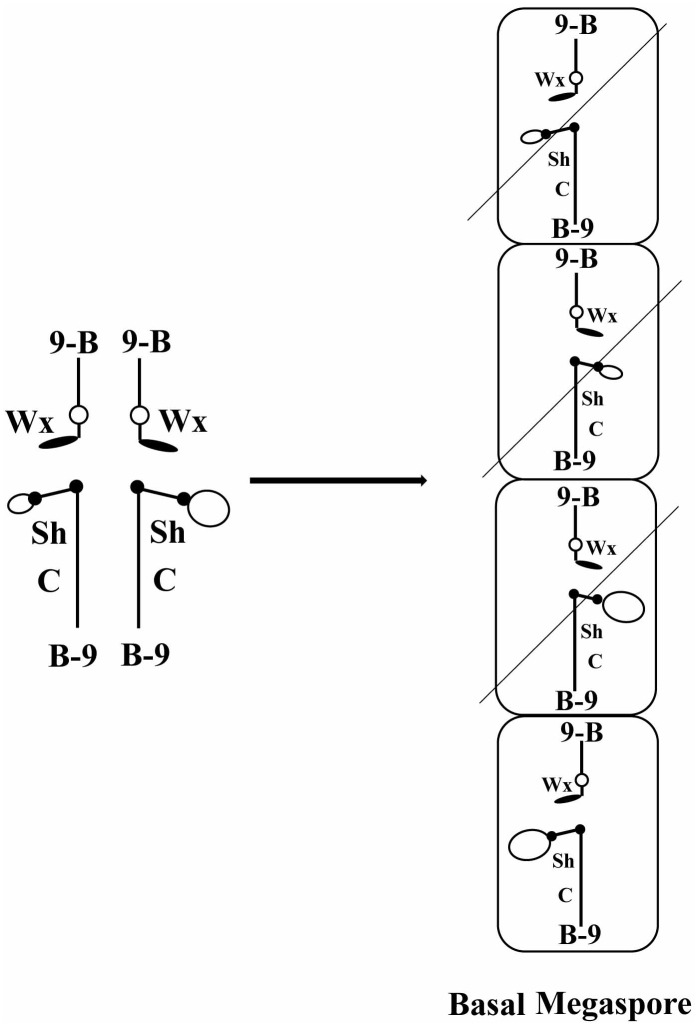
Diagram of how meiotic drive would operate in a heterozygote of two centromeres. At the left is diagrammed the configuration of a heterozygote of TB-9Sb/misdivision derivatives of the same. Large and small open ovals represent the normal and misdivision centromere, respectively. At the right is diagrammed the megaspores resulting from meiosis. Only the basal megaspore proceeds to form the female gametophyte, which produces the egg. If larger centromeres were directed to the basal megaspore, they would be present in the progeny at a frequency greater than Mendelian predictions.

**FIGURE 2:**
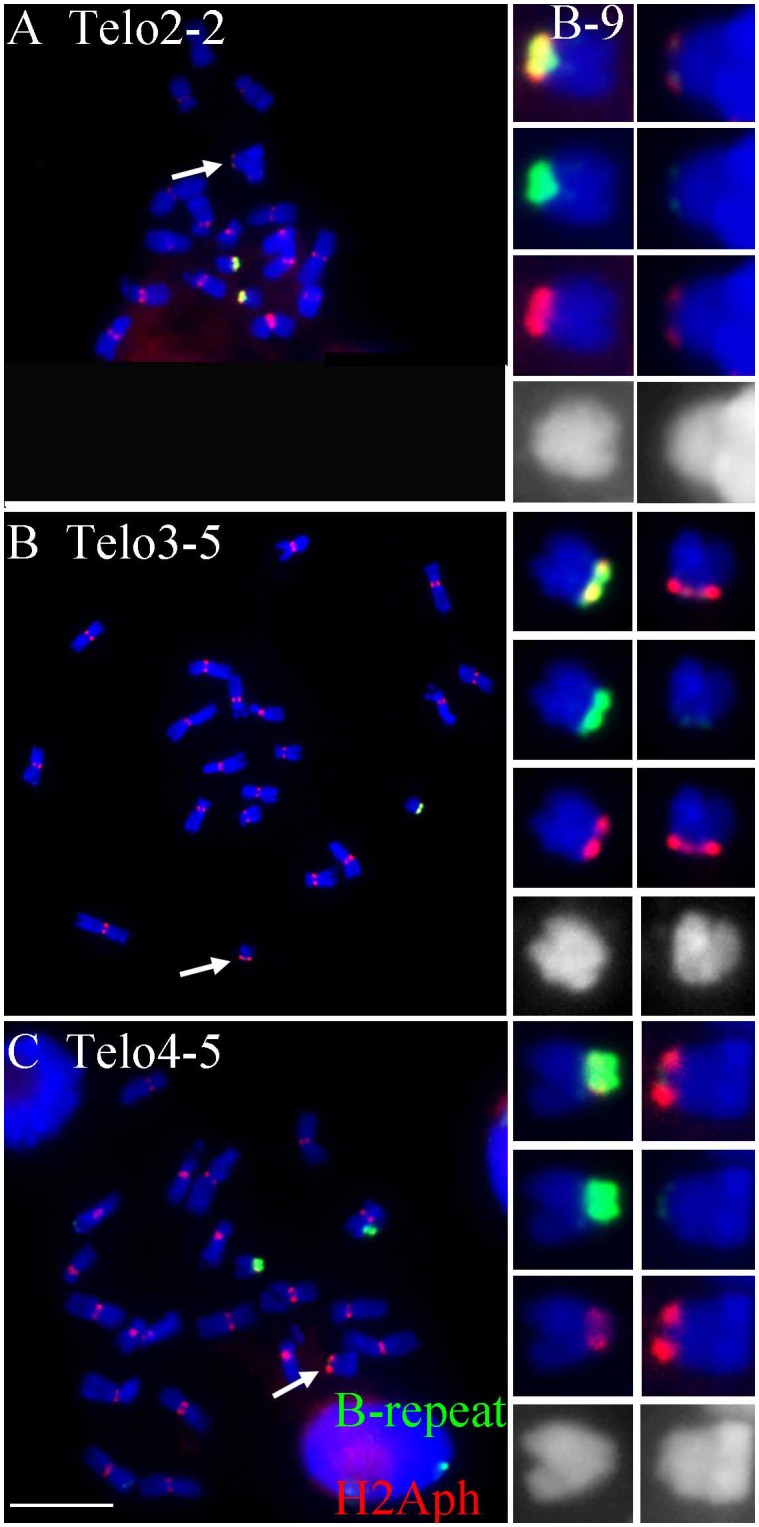
Comparisons of B specific repeat and H2Aph signals in heterozygotes of Telo 2-2 **(A)**, Telo 3-5 **(B)** and Telo 4-5 **(C)** with TB-9Sb, the progenitor chromosome. The B specific repeat is labeled in green and the label for H2Aph is red. In **A–C**, one misdivision chromosome is present, respectively (designated by arrows); **(A,C)** have two copies of the TB-9Sb, which contains the normal B centromere (green). Insets compare TB-9Sb normal (B-9) with the respective misdivision derivative. In the insets, the TB-9Sb chromosome is on the left and the respective misdivision chromosome is on the right; shown in descending order for each combination are composite, B specific (green), H2Aph (red) and gray scale for DAPI. Bar = 10 μm.

Toward this end, selected misdivision derivatives of TB-9Sb were self-pollinated in a pedigree until they were homozygous for the 9-B and B-9Sb chromosomes (with the understanding that the copy number of the B-9S chromosome can be variable in this stock because it undergoes non-disjunction at the second pollen mitosis). Then, four derivatives (Telo 3-5(+), Telo 2-2, Telo 4-5 and Telo 4-11) were crossed to homozygous TB-9Sb. Plants were selected that were homozygous for the 9-B chromosome, which is identical in all stocks, but heterozygous for the B-9Sb chromosomes (**Figure 1**). These plants were crossed as female by a tester stock for 9S, *c1 sh1 wx1*. The progeny of these crosses were germinated and individuals were examined in root tip cells for the presence of the large centromere (progenitor B-9Sb) or the small centromere (misdivision derivative of B-9Sb) based on the visible distinction of the FISH signal for the B chromosome centromere specific repeat, ZmBs (**Figure 2**). When both the large and small centromeres were found in a single individual in the progeny, these cases were recorded as female non-disjunction. The results are presented in **Table 2**.

**Table 2:** Determination of segregation ratio of large and small centromeres.

Derivative/TB-9Sb	Large	Small	NDJ	*X*^2^	*p*
Telo 3-5(+)	3054	2822	65	9.14	< 0.005
Telo 2-2	718	680	10	1.03	< 0.50
Telo 4-5	249	207	31	3.87	< 0.05
Telo 4-11	68	62	12	0.28	< 0.9


*The respective misdivision derivative paired with TB-9Sb are shown in the first column. The normal B centromere in TB-9Sb is designated “Large” and the misdivision centromere as “Small”. The number of seedlings in the segregating progeny of each type is shown. The number of seedlings in which both centromeres were recovered indicate non-disjunction (NDJ). Chi square and *p*-values are given for the null hypothesis of equal segregation.*

All of the four comparisons showed a higher numerical recovery of the larger centromere. Two of the four comparisons were significantly different in Chi Square tests (**Table 2**). Interestingly, the two that are significantly different also have the highest rates of non-disjunction in which both centromeres were recovered in a single individual. If the difference in segregation of the chromosomes with normal and reduced sized centromeres and the frequency of non-disjunction are related phenomena, it is worthy of note that the numbers for non-disjunction generally could account for the disparity of the large and small frequencies if they are added to the small class. Of course, the difference of segregation and the rate of non-disjunction are possibly unrelated.

### Estimation of the Size of the Biochemical Feature H2AphThr133 of Selected Misdivision Derivatives

To test whether the differences in transmission of the large and small centromeres correlated with the biochemical features of active centromeres, immunolocalization of a marker of active centromeres was performed on the three heterozygotes for which the greatest progeny sizes were assayed, Telo 3-5(+), Telo 2-2 and Telo 4-5. The phosphorylated form at Thr133 of Histone H2A has been shown to be a mark of active centromeres (Su et al., 2017). Heterozygotes were probed for H2Aph and with the B centromere specific repeat. **Figure 2** shows the results. In all three misdivision derivatives the amount of B specific repeat is barely detectable confirming the presence of the derivative. Telo 2-2 has a reduced H2Aph signal compared to TB-9Sb in the spreads illustrating that there is a correspondence between the amounts of centromeric DNA and biochemical features in this case. However, Telo 3-5(+) has an H2Aph signal that is comparable in size to TB-9Sb. Lastly, the H2Aph signal size for Telo 4-5 actually exceeds that of TB-9Sb. It is possible that the biochemical foundation of the kinetochore in these latter two cases expands similarly to how maize centromeres behave when introduced into oat (Wang et al., 2014). In the respective species, oat kinetochores are larger than maize, but when a maize chromosome is introduced into oat, the domain size expands (Wang et al., 2014). In the present study, there is a range of H2Aph quantities associated with the various active centromeres despite the small amount of centromeric DNA present in the three derivatives, which might indicate that in some cases the domain size of the centromere can expand if the centromere size is reduced.

### Segregation Strength Between the Normal B Centromere and Smaller Derivatives

#### Meiotic Analysis of TB-9Sb-Dp9 With Telo 2-2 in a Tug of War

**FIGURE 3:**
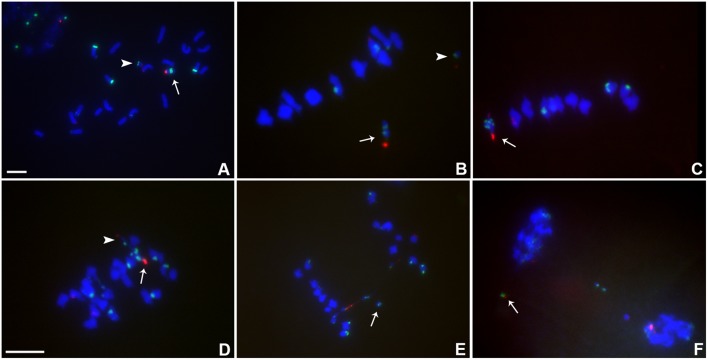
Cytological analysis of plants containing TB-9Sb-Dp9 and Telo 2-2. ZmBs, which is a B chromosome specific sequence in and around the centromere, is labeled in red. Knob heterochromatin is labeled in green. Chromosomes were counterstained with DAPI in blue. **(A)** Somatic cell, arrow indicates TB-9Sb-Dp9 chromosome and arrowhead denotes the Telo 2-2 chromosome. **(B)** Metaphase I, TB-9Sb-Dp9 does not pair with Telo 2-2 (arrow indicates TB-9Sb-Dp9; arrowhead indicates Telo 2-2). **(C)** Metaphase I. TB-9Sb-Dp9 paired with Telo 2-2 forming a bivalent (arrow). The large and small B centromeres (red) are directed to opposite poles. **(D)** Early anaphase I. An example of the large (arrow) and small (arrowhead) centromeres proceeding to different poles is shown (arrow). **(E)**. Anaphase I. A bridge was formed and an acentric fragment was released (arrow). **(F)**. Early telophase I. A bridge was formed and an acentric fragment was released (arrow). Bar = 10 μm.

From the collection of B chromosome centromere misdivision derivatives that have been described (Kaszas and Birchler, 1996, 1998), selected examples were used to cross with the plants containing the TB-9Sb-Dp9 chromosome. Plants containing TB-9Sb-Dp9 and Telo 2-2 were classified via FISH on root tip metaphase chromosomes using the B chromosome specific repeat (ZmBs) and knob heterochromatin (Peacock et al., 1981) probes. The TB-9Sb-Dp9 chromosome contains a large centromere and Telo 2-2 has a small one, which in comparison are distinct in cytological preparations (**Figure 3A**). Recombination in the 9S region between the foldback chromosome and the misdivision derivative occurs and forms a bridge as illustrated by a large and small centromere tied together in anaphase I (**Figure 3**).

The association in meiotic prophase between TB-9Sb-Dp9 and Telo 2-2 (+) is 60.43% (**Table 3**). There were 54.79% bridges formed in meiotic anaphase I (**Table 3**). The behavior of the two centromeres at various meiotic stages is shown in **Figure 3**. In the progeny of the heterozygotes of TB-9Sb-Dp9 and Telo 2-2, we did not find new dicentric chromosomes as occurred in the progeny of TB-9Sb-Dp9 with Telo 3-5(+) (Han et al., 2009).

**Table 3:** Meiotic analysis of hybrid plants containing TB-9Sb-Dp9 and Telo 2-2.

Pairing	No pairing	Total cells
55 (60.43%)	36 (39.56%)	91

**Anaphase I (bridge)**	**Anaphase I (no bridge)**	**Total cells**

40 (54.79%)	33 (45.21%)	73


*Five plants containing B-9Sb-Dp-9 and Telo 2-2 were used to observe meiosis for chromosome pairing and segregation. Pairing denotes when B-9Sb-Dp-9 and Telo 2-2 forms a bivalent and will form a bridge at anaphase I if exchange occurs followed by separation of the two centromeres to opposite during anaphase I.*

#### Meiotic Analysis of TB-9Sb-Dp9 With Telo 6-9 in a Tug of War

**FIGURE 4:**
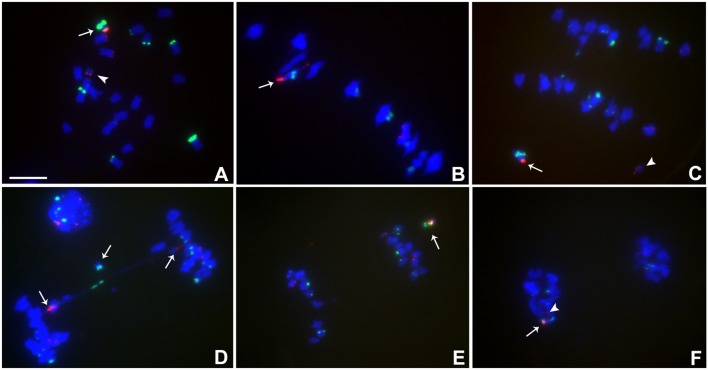
Cytological analysis of plants containing TB-9Sb-Dp9 and Telo 6-9. ZmBs is red; knob is labeled in green and chromosomes counterstained with DAPI. **(A)** Somatic cell, arrow indicates the TB-9Sb-Dp9 chromosome and the arrowhead indicates the Telo 6-9 chromosome. **(B)** Metaphase I. TB-9Sb-Dp9 pairs with Telo 6-9 (arrow). The two centromeres progress toward opposite poles. **(C)** Anaphase I. TB-9Sb-Dp9 (arrow) and Telo 6-9 (arrowhead) moved to the same pole and formed no bridge. **(D)** Anaphase I. An example of the large and small centromeres (arrows to red signals) proceeding to different poles is shown. A bridge was formed and an acentric fragment was released (arrow). **(E)** Anaphase I. TB-9Sb-Dp9 and Telo 6-9 moved to the same pole. Arrow indicates the TB-9Sb-Dp9 chromosome. **(F)** Telophase I. One telophase cell contained both red signals (arrow and arrowhead). Bar = 10 μm.

Heterozygotes of TB-9Sb-Dp9 and Telo 6-9 were identified as described above for other combinations. The behavior of the two centromeres at various stages is shown (**Figure 4A**). Recombination between the two chromosomes occurred, which produces bridges with a large and small centromere in anaphase I (**Figure 4**).

**FIGURE 5:**
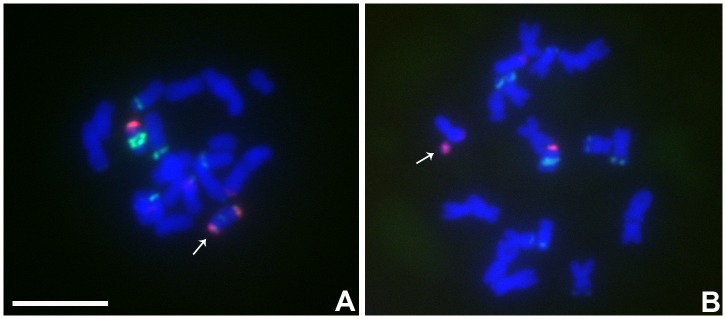
Cytological analysis the progeny from the plants contained TB-9Sb-Dp9 and Telo 6-9. ZmBs is red; knob is labeled in green and chromosomes counterstained with DAPI. **(A)** New dicentric chromosome was formed (arrow). **(B)** Fragment minichromosome with a B centromere in the progeny (arrow). Bar = 10 μm.

In this combination, examples of both centromeres progressing to the same pole were observed (**Figures 4C,E,F**) as well as cases in which the two centromeres progressed to opposite poles forming a bridge (**Figure 4D**). If recombination has occurred in those cases proceeding to the same pole, a dicentric would be produced. New dicentric chromosomes were detected in the progeny of these heterozygotes (**Figure 5**) as previously reported for the combination involving Telo 3-5(+) as the misdivision derivative (Han et al., 2009).

#### Meiotic Analysis of TB-9Sb-Dp9 With Telo 4-4, 4-5 and 4-11

**FIGURE 6:**
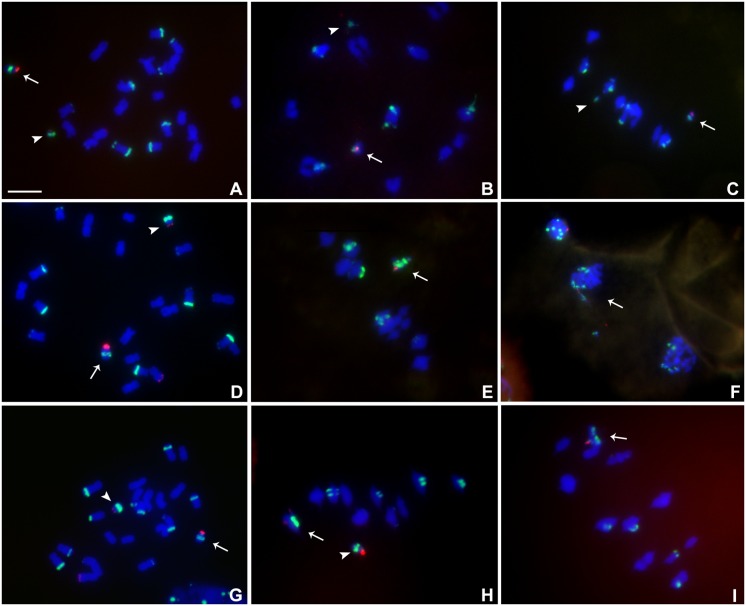
Cytological analysis of plants containing TB-9Sb-Dp9 and Telo 4-4, 4-5 and 4-11. ZmBs is labeled in red. Knob heterochromatin is labeled in green. Chromosomes were counterstained with DAPI in blue. **(A–C)** TB-9Sb-Dp9 (arrow) and Telo 4-4 (arrowhead), Somatic cell **(A)**. Diakinesis **(B)**. Metaphase I **(C)**. **(D)** Somatic cell FISH shows TB-9Sb-Dp9 (arrow) and T4-5 (arrowhead). **(E)** Metaphase I. TB-9Sb-Dp9 paired with Telo 4-5 (arrow). **(F)** Telophase I. A bridge and fragment were produced but the smaller centromere appears to be dragged to the same pole as the larger (arrow). **(G)** Somatic cell of TB-9Sb-Dp9 (arrow) and Telo 4-11(arrowhead). **(H)** Metaphase I. Telo 4-11 paired with chromosome 9 and 9-B forming a trivalent, consisting of the Telo4-11, 9-B and 9 chromosomes. Arrowhead indicates the separate TB-9Sb-Dp9 chromosome. **(I)** Metaphase I. Arrow indicates the TB-9Sb-Dp9 chromosome paired with T4-11(arrow). Bar = 10 μm.

Heterozygotes of TB-9Sb-Dp9 with Telo 4-4, Telo 4-5 or Telo 4-11 were identified using FISH probes as noted above for other combinations (**Figures 6A–I**). Telo 4-4, Telo 4-5 and Telo 4-11 are all further derivatives from misdivision chromosome Iso3 (-) (Kaszas and Birchler, 1998). **Figure 6F** shows an example in which the smaller centromere loses the tug of war and remains attached to the large centromere that has achieved migration to the telophase pole. With these noted exceptions, segregation of the large and small chromosomes occurs regularly.

## Discussion

The test of segregation of large versus small centromeres showed a skew toward greater recovery of the larger centromere in all four comparisons but this difference was only significant in two cases. The difference in the amount of centromeric DNA of the assayed examples is much greater than is likely to occur naturally but the segregation skew is not dramatic. Nevertheless, only a small difference in function, compounded over generations, could potentially drive centromere changes. However, as noted, the lower rate of recovery of the small centromeres could potentially be accounted for by non-disjunction if the small centromere entered the same product of meiosis I as the large centromere. While non-disjunction is distinct from meiotic drive, such a regular outcome of large/small centromere segregation could have a potential influence on centromere evolution.

However, such interpretations are complicated by the lack of a relationship of centromere size to the corresponding biochemical features as assayed in this study by determining the signal of phosphorylated histone H2A (Su et al., 2017). The combination (Telo 2-2/TB-9Sb) that produced a segregation that was normal had the greatest difference in H2Aph signal, which was greatly reduced in parallel to the DNA. The two comparisons that were statistically significantly different from a normal segregation (Telo 3-5 and Telo 4-5) had comparable or greater amounts of H2Aph signal associated with the smaller centromeres. Thus, there is not clear support for the concept of conflict between the size of the centromeric DNA and the accumulation of biochemical features of active centromeres (as assayed here by H2AphThr133). As noted, the sizes of the centromere differences are great but the departure from normal segregation rates, even though significant, are small. When coupled with the extensive evidence for epigenetic factors involved with centromeres noted above, a conclusion that there is an antagonism between centromere DNA and the kinetochore proteins is not straightforward.

In the centromere “tug of war” during meiosis, the large and small centromeres are tied together by recombination but they usually progress to opposite poles in anaphase I. Occasionally, they were included in the same anaphase I pole. In still other cases, the large and small centromeres appear to have attached to opposite poles but nevertheless appear destined to be included in the same nucleus. When this occurs, a dicentric is now present that can initiate the BFB cycle in subsequent cell divisions. Indeed, in the TB-9Sb/Telo6-9 combination, dicentrics were recovered in the next generation. In a previous study, one example (Dicentric-15) that was recovered was studied in detail (Han et al., 2009). This chromosome has the large and small centromere together as a structural dicentric. However, only the large centromere was associated with CENH3 and was active, while the small centromere was devoid of CENH3 and was inactive (Han et al., 2009).

In general, the large and small centromeres are effective in progressing to the poles in opposition. In some few cases, the smaller chromosome is extended from the pole with the larger chromosome and likely eventually becomes included in the same nucleus. In the case of Dic-15, the smaller centromere had become inactive. While chromosomes with similar structures were observed in meiosis and in the progeny of some combinations in this study, no determinations of centromere activity were made.

Here, studies of segregation frequency and strength were performed using a unique system in which different versions of a chromosome with varying sizes of centromeric DNA was used. While a slighter lower frequency of recovery of the small centromere was found in two combinations, there was no correlation with the phosphorylated form of histone H2A, which is a mark of active centromeres. When centromeres were placed in opposition to each other, they regularly segregated to opposite poles with minor exceptions.

Previous studies of misdivision derivatives as univalents showed a general correlation between centromere size and transmission frequency (with some exceptions) (Kaszas and Birchler, 1998). In this study, when representatives of this collection were placed in opposition to the progenitor TB-9Sb chromosome, the segregation was close to Mendelian predictions with a slight favor to the larger centromere in some combinations. The presence of a pairing partner appears to improve the transmission rate. Furthermore, the quantities of H2Aph on two of the three small centromeres was equivalent or greater than the progenitor chromosome centromere. This observation suggests that partial deletions of centromere DNA under natural conditions could potentially be of little consequence if the centromeric biochemical domain expands. The epigenetic flexibility of the centromere suggests that lesions to the underling DNA is not necessarily a major factor in centromere evolution.

## Author Contributions

JB, JL, and FH conceived the experiments. FH, JL, MM, ZG, and NS conducted the experiments. BZ performed the immunolocalizations. JB and FH wrote the paper.

## Conflict of Interest Statement

The authors declare that the research was conducted in the absence of any commercial or financial relationships that could be construed as a potential conflict of interest.
